# The gut–kidney microbiome–oxalate axis in calcium oxalate nephrolithiasis: mechanisms and microbiome-based interventions

**DOI:** 10.3389/fcimb.2026.1804800

**Published:** 2026-04-15

**Authors:** Shuo Pang, Zhenwei Zhang, Qifeng Ma, Yuxin Liu, Shiming Wang, Jianbo Wang, Yingwei Bi

**Affiliations:** 1 Department of Urology, The First Affiliated Hospital of Dalian Medical University, Dalian, China; 2 Department of Urology, Jinan Third People’s Hospital, Jinan, Shandong, China; 3 Dalian Medical University, Dalian, China

**Keywords:** calcium oxalate nephrolithiasis, gut microbiota, microbiome-targeted interventions, oxalatemetabolism, urinary microbiota

## Abstract

**Introduction:**

Calcium oxalate nephrolithiasis is increasingly recognized as a disorder influencednot only by diet and host oxalate handling, but also by the gut–kidneymicrobiome axis. Emerging multi-omics studies suggest that disturbances inintestinal and urinary microbiota, together with altered microbial metabolites,may contribute to disrupted oxalate homeostasis, inflammatory signaling, epithelialinjury, and crystal retention.

**Methods:**

We performed a narrative, semi-structuredreview of PubMed, Embase, and Web of Science (2010–2025), focusing onoxalate metabolism, gut and urinary microbiota, and microbiome-targeted interventionsin nephrolithiasis, with emphasis on calcium oxalate stones. Human andexperimental studies examining microbial composition, microbial metabolites,host transport and genetic determinants, and nutritional or microbial therapieswere qualitatively synthesized.

**Results:**

Current evidence indicates that loss of oxalatedegradinggut bacteria and broader dysbiosis are associated with hyperoxaluriaand increased calcium oxalate stone risk, whereas microbiome-supportive dietarypatterns may be protective. Multi-omics analyses reveal coordinated alterationsacross stool, urine, and stone-associated microbiota, implicating pathways involvingshort-chain fatty acids, bile acids, and unconjugated bilirubin in oxalatehandling, inflammation, and lithogenesis. Nutritional modulation may favorablyinfluence this axis, while probiotics, synbiotics, and engineered livebiotherapeutics show encouraging preclinical results.

**Discussion:**

Fecal microbiota transplantationremains highly preliminary in this field, and overall human data remainlimited and heterogeneous. The gut–kidney microbiome–oxalate axis providesan integrative framework linking diet, host pathways, microbial metabolites, andmulti-site microbial communities to calcium oxalate nephrolithiasis, and may helpinform future microbiome-based prevention and adjunctive managementstrategies.

## 1 Introduction

Nephrolithiasis (kidney stone disease) is a common and recurrent condition affecting approximately 10% of the global population, with incidence rising in parallel with lifestyle diseases such as obesity and metabolic syndrome (Wang et al., 2021; Miller et al., 2022). Calcium oxalate stones are the most prevalent type, accounting for >80% of cases. Traditional risk factors for calcium oxalate urolithiasis include high oxalate intake (e.g. from spinach, nuts), low dietary calcium, high animal protein and salt consumption, dehydration, obesity, and certain intestinal disorders that increase oxalate absorption. These factors all contribute to elevated urinary supersaturation with calcium and oxalate – the physicochemical driver of crystal formation. However, the precise mechanisms linking these risk factors to stone formation remain incompletely understood (Wang et al., 2021). In particular, there is growing recognition that oxalate homeostasis is not determined by the host alone but is strongly influenced by the gut microbiota and possibly the urinary microbiota (Miller et al., 2022; Sadaf et al., 2017; Assimos, 2012; Tian et al., 2022; Shen et al., 2021).

Historically, the role of microorganisms in nephrolithiasis was thought to be confined to *infection stones* (struvite calculi) caused by urease-producing pathogens. Today, emerging evidence indicates a much broader “gut–kidney axis” whereby intestinal microbes affect systemic metabolism and urinary composition, thus modulating kidney stone risk (Miller et al., 2022). The human intestine harbors bacteria capable of degrading oxalate or altering its absorption, such as *Oxalobacter formigenes*, and these microbes may protect against hyperoxaluria – a major risk factor for calcium oxalate stones (Wang et al., 2021; Whitaker et al., 2025). Conversely, perturbations of the gut microbiota (dysbiosis) might promote lithogenesis by increasing oxalate absorption and by generating pro-inflammatory or crystallization-promoting metabolites (Wang et al., 2021; Miller et al., 2022). Adding another layer of complexity, the urinary tract itself is now known to host a low-biomass urinary microbiota (also termed the “urobiome”), ending the dogma of sterile urine. Intriguingly, certain urinary commensal bacteria have been implicated in either inhibiting or accelerating stone crystallization within the kidney (Miller et al., 2022; Agudelo et al., 2024a; Wu et al., 2023).

Given these developments, the concept of a “gut–kidney microecology” has emerged as a novel paradigm in nephrolithiasis research. In this review, we comprehensively examine how the gut and urinary microbiota interact with oxalate metabolism and stone formation, integrating insights from multi-omics studies and mechanistic experiments. We first outline the normal pathways of oxalate metabolism and excretion, then discuss the evidence linking gut dysbiosis to kidney stone risk. We next explore the contribution of the urinary microbiota to stone pathogenesis and the metabolic crosstalk between gut and kidney microbes. Multi-omics investigations are highlighted to illustrate system-level changes in stone formers. Finally, we review translational strategies targeting the microbiome – from diet and probiotics to fecal transplantation and engineered bacteria – that aim to prevent or treat kidney stones. Throughout, we critically assess the current evidence, unresolved controversies, and future directions in this rapidly evolving field. Understanding the gut–kidney axis in oxalate metabolism may pave the way for innovative microbiome-based interventions in nephrolithiasis (Agudelo et al., 2024b).

Compared with previous narrative and systematic reviews that have typically examined oxalate homeostasis, the intestinal microbiota, or the urinary microbiota in isolation (Miller et al., 2022; Ticinesi et al., 2020; Bostanghadiri et al., 2021; Jung et al., 2023), the present review provides an integrated gut–kidney microbiome–oxalate axis framework for calcium oxalate nephrolithiasis. The objective of this review is to synthesize current evidence on host determinants of oxalate handling, gut and urinary microbiota alterations, and mechanistic insights emerging from multi-omics studies, and to connect these findings with microbiome-targeted preventive and therapeutic strategies, including dietary modulation, probiotics, fecal microbiota transplantation, and engineered live biotherapeutics. By organizing these data into conceptually linked schematics and summary tables, we aim to offer a translational roadmap that complements and extends prior reviews, and to provide clinicians and researchers with a practical framework for microbiome-informed prevention and management of calcium oxalate nephrolithiasis.

## 2 Methods

### 2.1 Literature search strategy

We conducted a narrative, semi-structured literature review focusing on the gut–kidney axis, oxalate metabolism, and microbiome-based interventions in nephrolithiasis. A comprehensive search of PubMed/MEDLINE, Embase, and Web of Science was performed for articles published between 2010 and 2025 (search last updated in 2025). The following combinations of keywords and Medical Subject Headings (MeSH) were used: “kidney stone*”, “nephrolithiasis”, “urolithiasis”, “calcium oxalate”, “hyperoxaluria”, “oxalate metabolism”, “oxalate transporter”, “SLC26A6”, “gut microbiota”, “intestinal microbiota”, “urinary microbiota”, “urobiome”, “microbiome”, “multi-omics”, “metabolomics”, “metagenomics”, “fecal microbiota transplantation”, “fecal microbiota transplantation (FMT)”, “probiotic*”, “synbiotic*”, and “engineered bacteria”. Reference lists of key primary studies and reviews were manually screened to identify additional relevant publications. For consistency, we use the terms “gut microbiota” and “urinary microbiota” throughout this review when referring to the microbial communities relevant to nephrolithiasis. The term “urobiome” is mentioned only at first reference for clarification and is not used thereafter.

### 2.2 Study selection process

**Figure 1:**
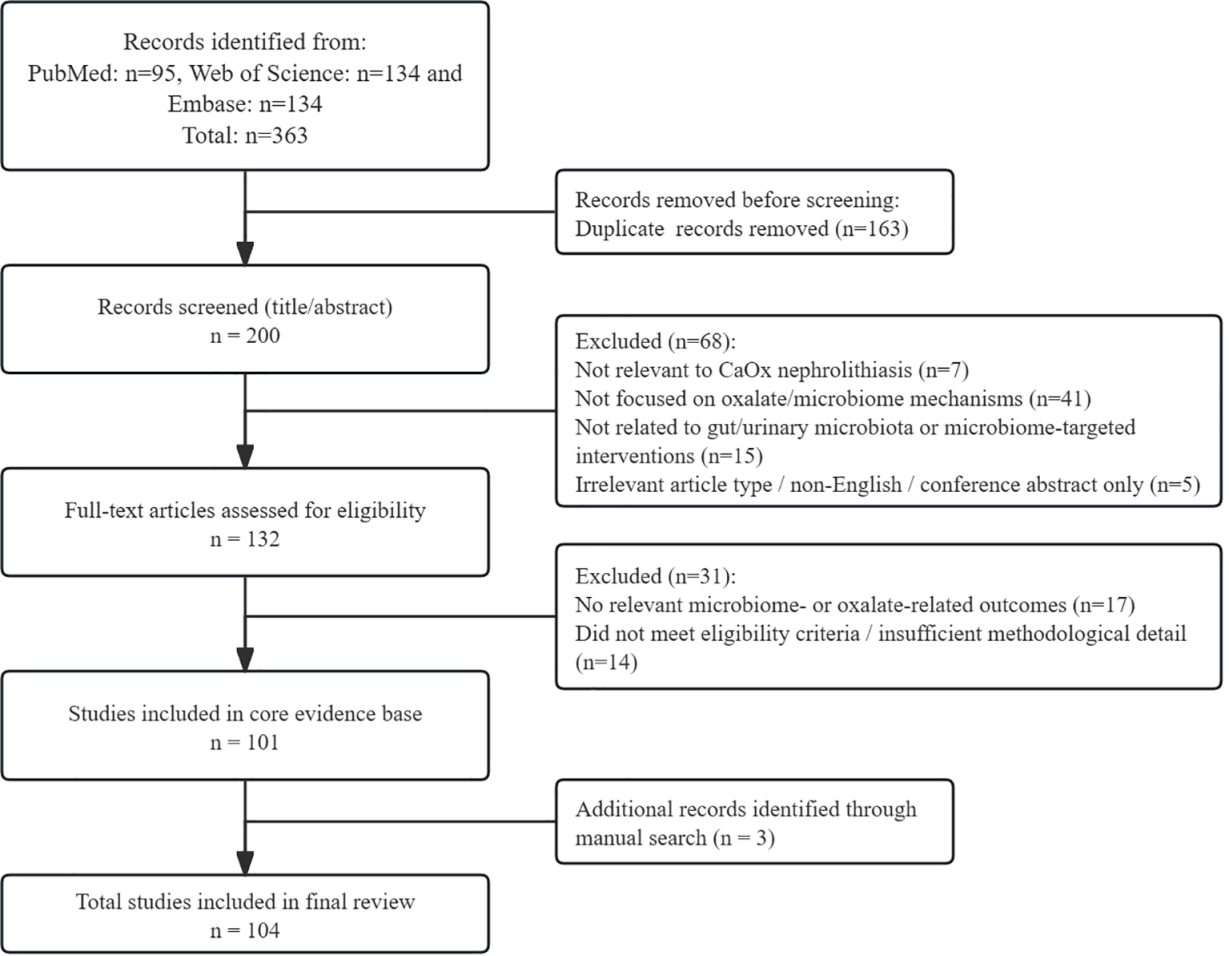
PRISMA-style flow diagram of literature identification, screening, eligibility assessment, and inclusion for this narrative, semi-structured review.

To improve reporting transparency, the literature selection process was structured in a Preferred Reporting Items for Systematic Reviews and Meta-Analyses (PRISMA)-aligned manner. A total of 363 records were initially identified through database searching, including 95 from PubMed/MEDLINE, 134 from Embase, and 134 from Web of Science. After removal of duplicates, 200 records remained for title and abstract screening. Of these, 68 records were excluded at the title and abstract stage. The remaining 132 articles underwent full-text assessment against the predefined inclusion and exclusion criteria. After full-text review, 31 articles were excluded because they did not sufficiently address the scope of the review, lacked relevant microbiome- or oxalate-related outcomes, or did not meet the predefined eligibility criteria. Ultimately, 101 studies were included in the core evidence base of this review. During revision, 3 additional relevant studies identified through manual screening and reviewer-suggested literature were incorporated to strengthen specific thematic sections, yielding a final total of 104 included references. The study selection process is summarized in **Figure 1**.

### 2.3 Study selection and eligibility criteria

To improve methodological transparency, the review question was refined using a Population, Intervention/Exposure, Comparator, Outcomes, and Study design (PICOS)-informed framework. The populations of interest included patients with calcium oxalate nephrolithiasis, individuals with hyperoxaluria, and relevant experimental animal models of stone formation or oxalate dysregulation. The main exposures and interventions considered in this review included gut and urinary microbiota composition and function, microbial metabolites, dietary modification, probiotics, synbiotics, fecal microbiota transplantation, and engineered microbial therapies. Comparators included healthy controls, non-stone formers, baseline conditions, or untreated groups where applicable. Outcomes of interest included urinary oxalate excretion, stone events, renal crystal deposition, microbiome composition, microbial metabolite changes, and host oxalate-handling mechanisms. Eligible study designs included observational studies, mechanistic investigations, animal experiments, and interventional trials. We included peer-reviewed original studies and selected narrative or systematic reviews that were directly relevant to the scope of this manuscript. We excluded case reports, conference abstracts without sufficient methodological detail, non-peer-reviewed opinion pieces, and articles not available in English.

### 2.4 Data extraction and synthesis approach

Given the heterogeneity in study design, populations, microbiome assessment methods, and reported outcomes, no formal meta-analysis was undertaken. Instead, we qualitatively extracted information on study type, sample size, population characteristics, microbiome sampling sites (stool, urine, stone surface), sequencing or analytical platforms (16S rRNA gene sequencing, shotgun metagenomics, metabolomics), key microbial taxa and metabolites, and clinical or biochemical endpoints relevant to oxalate homeostasis and stone formation. Evidence was synthesized narratively and organized into thematic domains: (i) gut microbiota alterations in stone formers; (ii) urinary microbiota characteristics and its interaction with crystals; (iii) multi-omics insights into host–microbe oxalate metabolism; and (iv) microbiome-based interventions and their translational potential. Wherever possible, we qualitatively considered the strength of evidence by taking into account study design (observational vs. interventional; human vs. animal), sample size, adjustment for confounding factors, and reproducibility across independent cohorts (Ma et al., 2021).

## 3 Gut–kidney axis and oxalate metabolism: an overview

Oxalate Homeostasis: Oxalate is a dicarboxylic acid end-product of metabolism that must be excreted, primarily via the kidneys. It has no known human physiological function, yet when present in high concentration it readily crystallizes with calcium to form calcium oxalate. Oxalate originates from two main sources: dietary intake and endogenous production (mostly from hepatic metabolism of glyoxylate). Under normal conditions, about 10–50% of urinary oxalate is estimated to come from the diet, with the remainder produced metabolically (Balawender et al., 2024; Crivelli et al., 2020). The intestines play a dual role in oxalate homeostasis – they are sites of oxalate absorption *and* secretion. In the upper gastrointestinal (GI) tract (small intestine), oxalate can be absorbed into the bloodstream, especially if dietary calcium is low (since calcium binds oxalate in the gut lumen). In the colon, oxalate can be secreted from blood into the lumen, providing an important route for oxalate elimination aside from renal excretion (Stepanova, 2023). This active enteric secretion is mediated by transporters such as SLC26A6 (also known as PAT1), an anion exchanger expressed on the apical membrane of intestinal epithelial cells. SLC26A6 exchanges luminal chloride for intracellular oxalate, thereby pumping oxalate into the gut lumen for fecal excretion (Stepanova, 2023). Animal studies demonstrate the significance of this pathway: mice lacking the SLC26A6 gene have greatly reduced fecal oxalate output and correspondingly elevated urinary oxalate, leading to hyperoxaluria and calcium oxalate stone formation (Stepanova, 2023). Thus, the host’s genetic and physiological capacity for intestinal oxalate secretion is a key determinant of stone risk. Certain diseases exemplify this gut–kidney axis: patients with inflammatory bowel disease or short bowel syndrome often develop “enteric hyperoxaluria” due to impaired gut barrier/transport function and altered microbiota, resulting in increased oxalate absorption and nephrolithiasis risk (Stepanova, 2023). These observations underline that individual variation in oxalate transport and metabolism can predispose to stones, independently of diet (Mehta et al., 2016; Noonin and Thongboonkerd, 2024; Ormanji et al., 2020; Liu et al., 2025d).

Role of Gut Microbiota in Oxalate Metabolism: Alongside host transport mechanisms, the gut microbiota profoundly influences oxalate balance. The colon is a major site where oxalate can be metabolized by bacteria. A classic example is *Oxalobacter formigenes*, an anaerobic Gram-negative bacterium that uses oxalate as its sole energy source, degrading it into formate and CO_2_ (Whittamore and Hatch, 2021). This organism lives in the colon and effectively acts as an oxalate sink – consuming dietary or endogenously secreted oxalate and thereby reducing the amount available for absorption into the bloodstream. The activity of *O. formigenes* has been shown to promote net oxalate secretion from the blood to the gut, possibly by stimulating oxalate transporters like SLC26A6 in the colonic mucosa (Liu et al., 2025b). Other gut bacteria, including certain Lactobacillus and Bifidobacterium species, also possess oxalate-degrading enzymes and may contribute to oxalate turnover, though typically to a lesser extent than *O. formigenes* (Whittamore and Hatch, 2021). Under healthy conditions, a balance is achieved wherein a portion of dietary oxalate is degraded or expelled in feces (a process facilitated by the microbiota and enteric secretion), and the remainder is excreted by the kidneys. This prevents excessive systemic accumulation of oxalate (Joubran et al., 2024b; Haaskjold et al., 2015).

**Figure 2:**
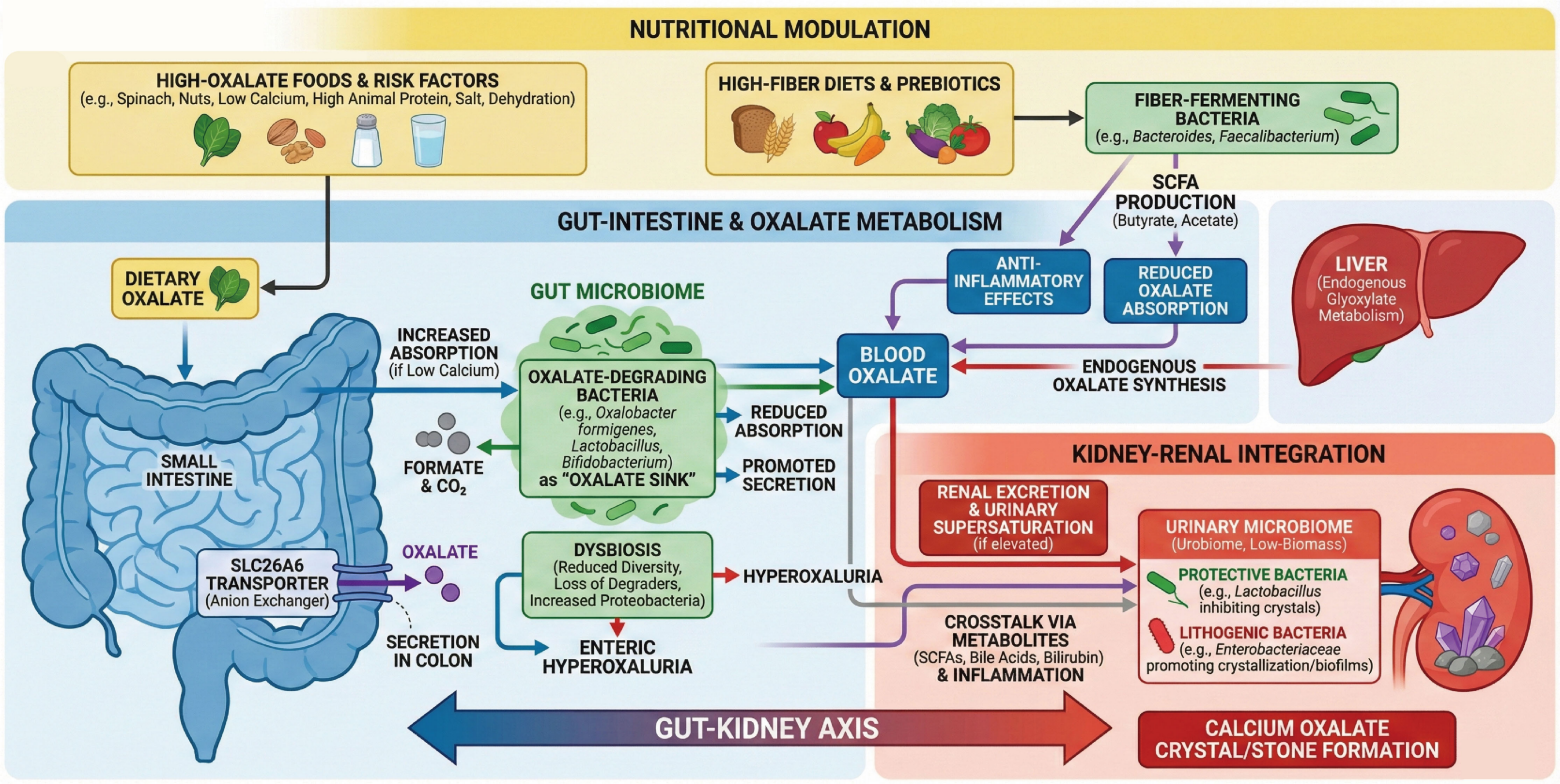
Schematic overview of the gut-kidney microbiome-oxalate axis in calcium oxalate nephrolithiasis. The diagram illustrates nutritional modulation (top), oxalate metabolism in the gut (center), microbial interactions and dysbiosis leading to hyperoxaluria, and kidney integration with urinary microbiota effects (bottom), highlighting key pathways, transporters (e.g., SLC26A6), metabolites (short-chain fatty acids (SCFAs), bile acids, bilirubin), and outcomes in crystal/stone formation.

Importantly, humans lacking oxalate-degrading gut bacteria have been observed to have higher urinary oxalate levels (Liu et al., 2025b). The gut microbiota can thus be viewed as an “oxalate filter” that offloads the excretory burden from the kidneys. This concept was first proposed decades ago but has gained considerable support from recent studies linking gut microbial composition to stone risk. The intimate connection between intestinal microbes and renal oxalate handling constitutes the core of the gut–kidney axis in oxalate metabolism (**Figure 2**) (Robijn et al., 2011; Wang et al., 2021). In subsequent sections, we delve into how disruptions of this axis – through microbial dysbiosis or antibiotic exposures – can tip the balance toward hyperoxaluria and stone formation.

## 4 Gut microbiota dysbiosis and kidney stone formation

### 4.1 Gut microbiota characteristics in kidney stone patients

Across case-control, pediatric, and meta-analytic datasets, the most reproducible gut signature in calcium oxalate stone formers is not a single taxon, but a broader dysbiotic configuration characterized by reduced microbial diversity, depletion of oxalate-degrading and short-chain fatty acid (SCFA)-producing taxa, and enrichment of potentially pro-inflammatory organisms. Thus, the central signal is a loss of functional resilience rather than one uniform compositional change across all cohorts. Representative examples include reduced α-diversity across adult and pediatric stone cohorts, distinct β-diversity clustering relative to healthy controls, depletion of fiber-fermenting genera such as Prevotella_9, and enrichment of taxa such as Bacteroides and Enterobacteriaceae in several datasets (Yuan et al., 2023; Zhao et al., 2021; Jaunet-Lahary et al., 2023; Gupta and Singh Kanwar, 2021; Al et al., 2023).

**Figure 3:**
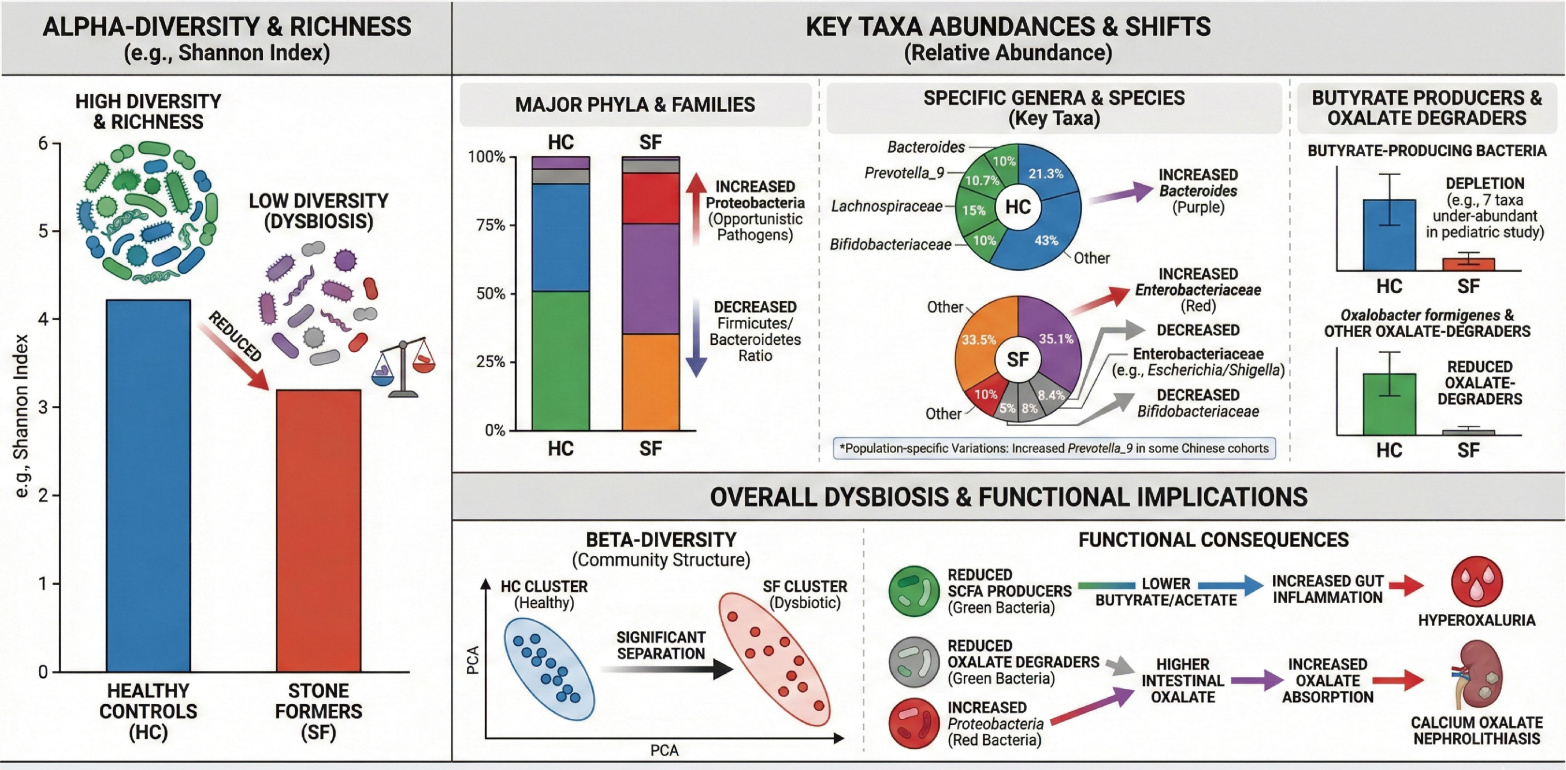
Comparative differences in gut microbiota composition between kidney stone formers (SF) and healthy controls (HC) in calcium oxalate nephrolithiasis. The figure depicts reduced alpha-diversity (top left), shifts in key taxa abundances (top center, e.g., increased Bacteroides, decreased Prevotella_9, Lachnospiraceae, Bifidobacteriaceae), depletion of butyrate producers and oxalate-degraders (top right, e.g., lower Oxalobacter formigenes), and overall dysbiosis indicators (bottom) with beta-diversity separation, reduced SCFA producers, increased Proteobacteria, and implications for inflammation, hyperoxaluria, and stone formation.

At the taxonomic level, stone formers often show enrichment of potentially dysbiotic taxa and depletion of beneficial commensals. The meta-analysis by Yuan et al. reported that the relative abundance of the genus *Bacteroides* was significantly higher in stone patients than in healthy controls (35.1% vs 21.3%; between-group comparison, P < 0.001), whereas the relative abundance of the fiber-fermenting genus Prevotella_9 was significantly lower in stone patients (8.4% vs 10.7%; between-group comparison, P < 0.00001) (Yuan et al., 2023). Several studies also report an increase in Enterobacteriaceae (e.g. *Escherichia/Shigella*) in the gut of stone formers (Yuan et al., 2023), alongside a reduction in key commensals that are thought to have health-promoting effects, such as certain Lachnospiraceae and Bifidobacteriaceae families (Al et al., 2023; Zhao et al., 2021). These shifts suggest a microbiota skewed toward a more inflammatory or pathogenic configuration in individuals with nephrolithiasis. Notably, the depletion of taxa that produce SCFAs like butyrate has been highlighted. In a pediatric study of early-onset stones, 7 out of 31 significantly under-abundant taxa in stone patients were butyrate-producing bacteria, indicating a loss of SCFA producers (Kim et al., 2022). This change in functional guilds may have downstream consequences for gut barrier integrity and systemic inflammation (discussed later). These differences in α-diversity, community structure and the relative abundance of key taxa between kidney stone formers and healthy controls are summarized schematically in **Figure 3** (Gnanandarajah et al., 2012).

It should be mentioned that not all studies find identical microbiota changes; some differences appear population specific. For instance, a study in a Chinese cohort by Zhao et al. observed a marked *increase* in *Prevotella_9* in stone patients relative to local healthy controls (8.8% vs 1.9%) (Zhao et al., 2021) – seemingly opposite to the aforementioned meta-analysis result. The same study also reported a dramatic increase in the ratio of phylum Bacteroidetes to Firmicutes (B/F) in stone patients (B/F = 0.67) compared to controls (B/F = 0.08) (Zhao et al., 2021), whereas other studies have not always noted such a shift. These discrepancies likely reflect geographic, dietary, or ethnic differences in gut microbiota, as well as different sequencing methodologies. Nonetheless, the common theme is that stone formers harbor a dysbiotic gut microbiota characterized by reduced beneficial bacteria and perturbed community structure (Yuan et al., 2023; Al et al., 2023). This dysbiosis might be both a consequence of stone disease (e.g. due to frequent antibiotic use or dietary changes) and an active contributor to its pathogenesis by altering oxalate metabolism.

### 4.2 Oxalate-degrading bacteria: O. formigenes and allies

Among the myriad gut microbes, those that catabolize oxalate are of special interest in kidney stone disease. Intestinal oxalate-degrading microbes are not limited to a single taxon but include several functionally relevant groups. The best characterized organism is *Oxalobacter formigenes*, an obligate oxalotroph that uses oxalate as its primary energy source. In addition, a range of lactic acid bacteria, including taxa now classified within *Lactiplantibacillus*, *Lacticaseibacillus*, *Limosilactobacillus*, and related genera, have been reported to exhibit oxalate-degrading activity to varying degrees. Some *Bifidobacterium* species may also contribute to oxalate turnover, although generally less efficiently than *O. formigenes*. Together, these observations suggest that intestinal oxalate degradation is supported by a broader microbial network rather than by a single dominant species.

Among these organisms, *Oxalobacter formigenes* remains the most extensively studied and best supported protective candidate in calcium oxalate stone disease. *O. formigenes* resides in the colon and uses oxalate as its energy source, effectively clearing oxalate from the gut lumen. Colonization with *O. formigenes* has been associated with significantly lower urinary oxalate excretion in both animal models and humans (Liu et al., 2025b). Epidemiological studies have repeatedly found that kidney stone patients are less likely to carry *O. formigenes* in their intestines compared to non-stone formers (Pebenito et al., 2019a). Its reduced prevalence in modern populations has been linked in part to repeated antibiotic exposure, supporting the idea that loss of endogenous oxalate degraders may contribute to lithogenic risk (Suryavanshi et al., 2025; Daniel et al., 2021; Tavasoli et al., 2020; Manna et al., 2020; Pebenito et al., 2019a; Barnett et al., 2016; Duncan et al., 2002; Sidhu et al., 1999).

Mechanistically, the absence of *O. formigenes* can lead to insufficient oxalate breakdown in the gut, so more dietary or endogenous oxalate remains available for absorption, ultimately raising the urinary oxalate load and promoting calcium oxalate supersaturation (Robijn et al., 2011). However, attempts to supplement *O. formigenes* in humans have yielded mixed results. Some early trials showed promise in reducing urinary oxalate, but others did not find a significant effect on oxalate excretion or stone recurrence (Bostanghadiri et al., 2021). Challenges include the bacterium’s fastidious anaerobic requirements and difficulty achieving sustained colonization in the complex gut ecosystem of humans. Recent insights indicate that *O. formigenes* works in concert with the host – for example, its presence may upregulate colonic secretion of oxalate via SLC26A6, amplifying oxalate clearance (Liu et al., 2025b). The inconsistent results of *O. formigenes* supplementation highlight the complexity of the gut–oxalate axis and suggest that a whole-community approach (rather than single-species) may be required to meaningfully lower urinary oxalate.

In addition to *O. formigenes*, several lactic acid bacteria have also been reported to exhibit oxalate-degrading activity, although generally less efficiently. It should be noted that the former genus Lactobacillus was reclassified after 2020 into 23 genera, and a number of these reclassified taxa are relevant in the context of oxalate metabolism and probiotic development. Representative examples include Lactiplantibacillus (formerly including *Lactobacillus plantarum*), Lacticaseibacillus (formerly including *Lactobacillus casei* and *Lactobacillus rhamnosus*), *Ligilactobacillus*, *Levilactobacillus*, and *Limosilactobacillus* (formerly including *Lactobacillus fermentum*), several of which have been reported to degrade oxalate or reduce oxalate availability in experimental settings (Whittamore and Hatch, 2021; Al-Kabe and Niamah, 2024, 2025). In addition, some Bifidobacterium species may also contribute to oxalate turnover. Notably, stone formers tend to have lower abundances of several of these oxalate-degrading lactic acid bacteria in their gut microbiota (Yuan et al., 2023; Bacchetta and Lieske, 2022). Antibiotic experiments in animals reinforce their importance: when mice were given oral antibiotics that eliminated much of the gut flora, they developed hyperoxaluria and were more prone to calcium oxalate (CaOx) crystal deposition (Robijn et al., 2011). Overall, the depletion of oxalate-degrading bacteria is one of the most consistent microbial features linked with kidney stones. However, current human evidence remains more consistent with association than definitive causation, and the protective role of these taxa likely depends on broader ecological and host context. Taken together, current evidence supports the view that maintenance of a gut microbiota rich in oxalate-degrading functions is beneficial for oxalate homeostasis, whereas depletion of these functions may predispose to hyperoxaluria and stone formation (Robijn et al., 2011; Pebenito et al., 2019a). The major gut oxalate-degrading taxa discussed above, together with their oxalate-degradation capacity, prevalence patterns, antibiotic susceptibility and current clinical development status, are summarized in **Table 1** (Miao et al., 2025; Miller et al., 2019).

**Table 1:** Key oxalate-degrading bacterial species and their characteristics.

Bacterial species	Oxalate degradation capacity	Prevalence in healthy vs stone formers	Antibiotic sensitivity	Clinical trial status
O. formigenes	High (obligate oxalotroph, degrades to formate/CO2 via oxc/frc genes)	Higher in healthy (31-77%), lower in stone formers (e.g., 17% vs 38%)	Sensitive to fluoroquinolones and many antibiotics	Ongoing phase 1/2a trials in healthy and PH1 patients (e.g., NCT06330246)
Lactiplantibacillus plantarum	Modest to high in strains (e.g., AR1089 degrades oxalate in rat models)	Common in gut; oxalate-degraders lower in stone formers	Variable; some strains resistant	Preclinical/rat studies; some human trials reducing urinary oxalate
Lactobacillus acidophilus	Modest; inhibits CaOx crystallization and adhesion	General probiotic; degraders lower in stone formers	Variable depending on strain	Part of probiotic mixtures in trials reducing urinary oxalate
Bifidobacterium spp.	Modest (e.g., B. animalis subsp. lactis degrades oxalate)	Higher in healthy; decreased in stone formers	Variable sensitivity	In mixtures with Lactobacillus, trials show reduced urinary oxalate
Engineered P. vulgatus	Engineered high degradation to formate/CO2	N/A (engineered, controlled colonization)	Not specified; designed for stability	Phase 1/2a trial (Whitaker et al., 2025); reduced urine oxalate in models and humans

CaOx, calcium oxalate; PH1, primary hyperoxaluria type 1; FMT, fecal microbiota transplantation; HC, healthy controls; SF, stone formers; N/A, not applicable.

Oxalate degradation capacity is summarized qualitatively based on *in vitro* and *in vivo* data describing the ability of each taxon to metabolize oxalate (e.g., via oxc/frc-dependent pathways for *Oxalobacter formigenes*). Prevalence in healthy vs. stone formers reflects representative ranges reported in cohort studies, in which *O. formigenes* and other oxalate-degrading taxa are more frequently detected in HC than in SF. Antibiotic sensitivity refers to typical patterns reported for each genus or species; actual susceptibility profiles are strain-dependent and may vary by region and exposure history. Clinical trial status indicates whether the taxon has been evaluated as a probiotic or live biotherapeutic product in animal models or human studies (e.g., ongoing phase 1/2a trials of *O. formigenes* and engineered *Parabacteroides vulgatus* in PH1 or hyperoxaluric populations). Taxonomic names for lactic acid bacteria were updated where appropriate according to the post-2020 reclassification of the former genus Lactobacillus.

### 4.3 Other gut microbial alterations and stone phenotypes

In addition to oxalate degraders, other facets of gut microbiota imbalance may influence kidney stone risk or correlate with stone subtypes. One aspect is the overgrowth of bacteria that could indirectly promote stones. For example, an increase in certain Gram-negative Enterobacteriaceae in the gut (as often noted in stone formers (Yuan et al., 2023)) might contribute to systemic endotoxemia and inflammation, which in turn can alter hepatic oxalate metabolism or renal crystal retention (we discuss these inflammatory pathways later) (Wang et al., 2021). Some gut microbes produce metabolites (e.g. uricase-producing bacteria affecting uric acid levels, or ones influencing citrate metabolism) that might tilt the risk for different types of stones (calcium oxalate vs uric acid stones). Research in this area is still nascent, but there are hints that gut microbiota profiles might vary with stone composition.

A comparative analysis by Zhao et al. examined gut microbiota among patients with different stone types – calcium oxalate (COKS), uric acid (UAKS), and calcium phosphate (carbonate apatite) stones – versus healthy controls (Zhao et al., 2021). As mentioned, that study found general dysbiosis in all stone patients relative to controls (e.g. higher Bacteroidetes and lower beneficial genera like *Blautia* and *Lachnoclostridium* in stones) (Zhao et al., 2021). Interestingly, it also reported significant differences between stone types. Patients with uric acid stones had a higher abundance of *Prevotella_9* in their gut than those with calcium oxalate stones (Zhao et al., 2021). While the clinical implications are unclear, this suggests that specific microbial signatures might be associated with specific lithogenic pathways (for instance, *Prevotella* might be linked to diets rich in certain substrates common in hyperuricosuria). Another study noted that recurrent stone formers had some distinct microbiome features compared to first-time stone formers, implying that the microbiota might also relate to stone recurrence risk (Liu et al., 2020; Joubran et al., 2024a; Al et al., 2020; Wei et al., 2021; Liu et al., 2017).

Moreover, the reduction in SCFA-producing bacteria in stone patients is consistently observed and may be functionally relevant. SCFAs like butyrate, propionate, and acetate, produced by fermentation of dietary fibers by gut bacteria, have beneficial effects on intestinal health and systemic metabolism. Reduced butyrate producers (e.g. *Roseburia, Clostridium IV, Eubacterium*) have been linked to increased gut permeability and low-grade inflammation. In the context of stones, a 2020 multi-omics study found that stone formers did not necessarily have fewer oxalate-degradation genes in their gut microbiota, but they had a significant depletion of genes involved in SCFA production, along with lower fecal levels of SCFAs (Liu et al., 2020). In fact, the fecal acetic acid concentration was highest in patients with recurrent stones who also had high urinary oxalate and was positively correlated with genes for oxalate synthesis (Liu et al., 2020). This seemingly paradoxical finding (more acetate in high-oxalate patients) might reflect a compensatory response or dysbiosis-driven alteration in fermentation pathways. Notably, administration of SCFAs (like acetate) to hyperoxaluric rats in that study reduced renal crystal deposition (Liu et al., 2020), suggesting that SCFAs or the microbes that produce them could protect against stone formation, possibly by maintaining gut epithelial health or modulating urinary citrate excretion (Ferraro et al., 2019; Jin et al., 2021). These observations are further supported by experimental studies indicating that SCFAs can attenuate calcium oxalate crystal deposition through G protein-coupled receptor 43 (GPR43)-dependent immunomodulatory signaling and regulation of intestinal oxalate transport pathways.

Finally, even though most research focuses on calcium oxalate stones, gut microbiota changes have also been observed in other stone types. Uric acid stone patients, for example, may have distinct gut metabolic profiles (since uric acid stones are linked to obesity, insulin resistance, and low urine pH, factors that are known to be influenced by the gut microbiota). One study found that gut microbiota-derived metabolites differed between uric acid stone formers and calcium stone formers (Zhao et al., 2021), hinting at microbiome involvement in urine pH or purine metabolism. Likewise, in infection stones (struvite), while the primary cause is urinary infection, the gut can be a reservoir for urease-producing organisms such as *Proteus* that eventually cause urinary tract infections (UTIs). In short, various gut bacteria beyond just *O. formigenes* could contribute to a “pro-lithogenic” or “anti-lithogenic” milieu through metabolites like SCFAs, bile acids, or systemic inflammatory modulation. Deciphering these contributions requires integrated analysis, which we will explore in the multi-omics section. What is clear so far is that gut dysbiosis is a consistent feature of kidney stone patients, characterized by reduced diversity, loss of oxalate-degraders and SCFA-producers, and expansion of potentially harmful taxa (Yuan et al., 2023; Al et al., 2023). This dysbiosis likely interacts with the host’s diet and genetic factors to influence stone propensity (Chmiel et al., 2023).

## 5 Urinary microbiota and kidney stones

### 5.1 The urinary microbiota: concept and detection

The notion that urine is sterile in healthy individuals has been debunked in the past decade. Advanced DNA sequencing and enhanced culture techniques have revealed that the urinary tract harbors a resident microbial community, referred to here as the urinary microbiota, even in the absence of overt infection. This microbiota exists at relatively low biomass and can be difficult to characterize due to contamination risks, but its presence is now accepted. The discovery of the urinary microbiota opens new questions about its role in urological diseases, including nephrolithiasis (Miller et al., 2022; Jung et al., 2023; Xu et al., 2022).

Detecting and studying the urinary microbiota pose unique challenges. Routine midstream urine specimens may pick up contaminants from the lower urinary tract; thus, some studies use catheterized urine, suprapubic aspirates, ureteral urine, or stone-associated biofilm sampling to more directly assess kidney and bladder microbiota (Jung et al., 2023). Methods include 16S rRNA gene sequencing, metagenomic shotgun sequencing, and specialized culture, such as expanded quantitative urine culture. Each approach has limitations: 16S can be prone to background noise at low bacterial loads, while culture may miss uncultivable organisms. Moreover, stringent controls are required to distinguish true urinary microbes from laboratory contaminants. Due to these challenges, different studies have sometimes reported inconsistent urinary microbiota compositions. For example, some have detected lactic acid bacteria, including Lactobacillus, as dominant urinary taxa in healthy women, whereas others have reported relatively low abundances of these organisms and instead identified Corynebacterium or Staphylococcus as prevalent commensals (Jung et al., 2023). These discrepancies underscore the need for standardized protocols in urinary microbiota research (Jung et al., 2023). To improve reproducibility in future urinary microbiota studies, standardized protocols should ideally include the use of low-contamination sampling methods, such as catheterized urine or suprapubic aspirates where appropriate, together with rigorous negative controls at the stages of collection, DNA extraction, library preparation, and sequencing. Uniform handling of urine volume, processing time, storage conditions, and freeze–thaw exposure should also be specified in advance and consistently reported. In low-biomass settings, sequencing results should preferably be interpreted alongside absolute microbial load measurements and, where feasible, validated with complementary culture-based approaches such as expanded quantitative urine culture. Standardized bioinformatic decontamination workflows, batch randomization, and transparent reporting of contamination filtering criteria will also be essential to distinguish true urinary signals from background noise and to enable meaningful comparisons across studies. Nevertheless, there is now broad agreement that a baseline microbial community exists in urine and that it can shift in disease states.

In the context of kidney stones, initial studies suggest that stone formers have an altered urinary microbiota compared to non-stone formers (Al et al., 2023; Jung et al., 2023). However, given the low microbial abundance, distinguishing true signals has been difficult. It is also possible that bacteria associated with stones might predominantly exist in biofilms on stone surfaces or renal tissue rather than free-floating in urine. In one study that performed 16S sequencing on surgically obtained kidney stone samples, a variety of bacteria were found embedded in the stone matrices (Al et al., 2023). As research progresses, improved techniques (like sequencing after extensive urine centrifugation or using catheterized urine) should clarify the composition of the urinary microbiota and reduce false signals. For now, we recognize that the urinary tract is not sterile and that these resident microbes – albeit few – could influence processes like crystal nucleation, growth, or prevention thereof. Below, we summarize known examples of how the urinary microbiota may affect stone pathogenesis.

### 5.2 Impact of urinary microbes on stone formation

The most direct link between microbes and kidney stones is seen in infection stones, which are composed of struvite (magnesium ammonium phosphate) and carbonate apatite. Infection stones result from colonization of the urinary tract by urease-producing bacteria (classically *Proteus mirabilis*, but also *Klebsiella, Pseudomonas*, or urease-positive *E. coli*). Bacterial urease hydrolyzes urea into ammonia, alkalinizing the urine (raising pH) and providing the ingredients for struvite precipitation. This mechanism has been well known for decades (Jung et al., 2023). In this setting, the urinary microbiota is overtly pathogenic, and infection control is central to management.

What about non-infection stones like calcium oxalate stones? Even here, the urinary microbiota might play subtle roles in modulating stone formation. One intriguing study by Agudelo et al. demonstrated direct effects of altering the kidney’s resident bacteria on CaOx crystallization (Agudelo et al., 2024a). In a mouse model, researchers administered the antibiotic cefazolin, which was found to selectively deplete *Lactobacillus* species in the urinary tract while allowing gram-negative *Enterobacteriaceae* (like *E. coli*) to proliferate in kidney tissue (Agudelo et al., 2024a). The result was a significantly increased deposition of CaOx crystals in the kidneys of antibiotic-treated mice compared to controls (Agudelo et al., 2024a). Conversely, when the mice were supplemented with a probiotic *Lactobacillus* strain, renal crystal formation was suppressed, whereas introduction of an *E. coli* isolate enhanced crystal accumulation (Agudelo et al., 2024a). This elegant experiment provides proof-of-concept that shifting the balance of urinary microbes can directly influence lithogenesis. Mechanistically, these effects are most plausibly mediated through changes in local urine chemistry, epithelial injury/inflammation, and crystal adhesion or biofilm-related interactions (Agudelo et al., 2024a) (Wang et al., 2021; Agudelo et al., 2024a; Jung et al., 2023; Dong et al., 2022; Ticinesi et al., 2019)).

Clinical observations support these findings. A recent review identified that *Lactobacillus* was more frequently found in the urine of healthy individuals, while stone patients’ urine tended to be enriched in *Enterobacteriaceae* and lacked beneficial lactobacilli (Jung et al., 2023). Additionally, metagenomic analysis of urine from CaOx stone patients revealed a decrease in microbial genes for oxalate degradation (which presumably could occur in urine or kidneys) and an increase in genes related to adhesion and inflammation in their urinary microbiota (Al et al., 2023; Bacchetta and Lieske, 2022). Together, these early clinical and metagenomic observations suggest that urinary microbial communities in stone formers may shift toward a more adhesive, pro-inflammatory, and potentially lithogenic functional profile (Zaidan et al., 2024; Berman and Merritt, 2018).

That said, research in this area is in its infancy. One major issue is reproducibility – different studies sometimes detect different organisms in urine, likely due to contamination or technical variability (Jung et al., 2023). Current evidence suggests that urinary microbes may influence lithogenesis through three non-exclusive mechanisms: altering local urine chemistry, interacting physically with crystals through adhesion or biofilm-related processes, and modulating epithelial injury or inflammatory responses (Agudelo et al., 2024a; Jung et al., 2023). In infection stones, these effects are extreme (high pH, etc.), but in calcium oxalate stones, they may be subtler yet still significant over the long term.

### 5.3 Interplay between gut and urinary microbiota

Rather than viewing the gut and urinary microbiota in isolation, an integrative perspective is emerging: the “gut–kidney axis” involves crosstalk between these two microbial communities that together influence stone risk (Miller et al., 2022). The gut microbiota influences the metabolite load delivered to the kidney, whereas the urinary microbiota may modify crystal behavior locally within the renal and urinary environment. These two ecosystems can also communicate indirectly through host-mediated pathways – for example, gut bacteria can trigger inflammatory or metabolic changes that affect the kidney environment.

Miller et al. describe a multi-level model in which the host’s various microbiomes collectively modulate lithogenesis (Miller et al., 2022). For instance, a dysbiotic gut microbiota with low oxalate-degrading capacity will deliver a higher oxalate load to the kidney, increasing supersaturation and stone risk. Simultaneously, if the urinary microbiota shifts toward a lithogenic profile (e.g. loss of *Lactobacillus* and overgrowth of lipopolysaccharide (LPS)-secreting Gram-negatives), the kidney environment becomes more permissive to crystals – through higher pH, biofilm formation on Randall’s plaques, or inflammation-mediated epithelial damage that traps crystals (Miller et al., 2022; Agudelo et al., 2024a). Thus, gut and urinary microbiota may act in concert either to promote or to restrain lithogenesis, depending on their metabolic and inflammatory profiles. This integrated view is supported by observations that stone formers often have concurrent dysbiosis in multiple body sites. Al et al. (2023) found that kidney stone patients had distinguishable microbiome profiles not only in stool but also in urine and even on retrieved stone surfaces, compared to non-stone-formers (Al et al., 2023). The changes spanned different body habitats, suggesting a systemic alteration in microecology in stone patients (Al et al., 2023). Similarly, another analysis in pediatric stone formers noted that lower gut microbial diversity correlated with lower urinary citrate (a known stone inhibitor) and higher urine oxalate – linking gut dysbiosis with urinary chemistry disturbances (Bacchetta and Lieske, 2022; Liu et al., 2021).

Taken together, these findings support a network-based view in which gut and urinary microbiota should not be considered independent contributors, but interconnected components of the gut–kidney microbiome–oxalate axis. Disruption at one end (gut) can have downstream effects on the other (urine), and vice versa, through metabolic and immunological links (Miller et al., 2022; Agudelo et al., 2024a).

## 6 Mechanistic insights from multi-omics studies

### 6.1 Oxalate metabolic pathways: host transport and genetic factors

A solid mechanistic understanding must begin with the host’s own oxalate metabolism, as it sets the baseline onto which microbial effects are layered. We discussed earlier the key role of the intestinal transporter SLC26A6 in mediating oxalate excretion. To reiterate, SLC26A6 on enterocytes actively secretes oxalate into the gut lumen, defending against hyperoxalemia and hyperoxaluria (Stepanova, 2023). Mice lacking SLC26A6 develop severe hyperoxaluria and calcium oxalate stones, establishing that impaired enteric oxalate secretion alone can cause nephrolithiasis (Stepanova, 2023). Interestingly, recent research shows that SLC26A6 deficiency doesn’t only affect oxalate transport – it also leads to changes in the gut microbiota and intestinal inflammation (Anbazhagan et al., 2024). Anbazhagan et al. (2024) demonstrated a direct link between loss of SLC26A6 and gut microbial dysbiosis, along with a compromised gut barrier and increased intestinal inflammation (Anbazhagan et al., 2024). The absence of the oxalate transporter likely results in oxalate retention in the gut, altering the niche and favoring oxalate-accumulating or other dysbiotic microbial communities, which in turn cause mucosal barrier damage and inflammation (Anbazhagan et al., 2024). This finding underscores a two-way interplay: host genotype can shape the microbiome (and inflammation status), which may then further exacerbate hyperoxaluria – a vicious cycle potentially. It suggests that individuals with certain genetic polymorphisms or deficiencies in oxalate handling (e.g. SLC26A6 variants) might be inherently prone to both dysbiosis and stones. Besides SLC26A6, other host factors include endogenous oxalate production. Primary hyperoxaluria (PH) is a group of rare genetic disorders where hepatic enzymes are deficient, leading to overproduction of oxalate. These patients invariably suffer from recurrent stones and kidney injury. While PH is rare, more common polymorphisms in enzymes like AGXT or GRHPR might subtly affect endogenous oxalate levels in the general population. The microbiome might serve as an extra buffer for such individuals (Zhu et al., 2020; Miller et al., 2017b).

Moreover, renal handling of oxalate merits attention. The kidneys filter oxalate freely and then may reabsorb a portion or secrete more into urine. Specific transporters in the renal tubules (SLC26A1, SLC26A6, and others) are thought to mediate tubular oxalate secretion, but this is less well characterized than gut transport. A concept worth noting is that the kidney itself might produce oxalate under certain pathological conditions (e.g. from vitamin C metabolism in renal cells), although this is minor compared to liver production (Wang et al., 2021). Ultimately, from a mechanistic viewpoint, the propensity for stones arises when urinary oxalate concentration persistently exceeds the threshold of CaOx supersaturation. This can occur due to: (a) excessive dietary or endogenous oxalate load relative to calcium, (b) inadequate intestinal excretion (genetic or microbial causes), or (c) excessive renal reabsorption of oxalate. Each of these has a host genetic component and a microbial component. Thus, multi-omics approaches that integrate host genomics, transcriptomics, and microbiomics are valuable to tease apart their contributions. For example, a recent integrative analysis attempted to incorporate host genotype and gut metagenome to predict kidney stone risk, finding that combining factors improved predictive power over either alone (Kim et al., 2022). Though in early stages, such systems biology approaches highlight that host and microbe factors jointly determine oxalate metabolic phenotypes.

### 6.2 Microbe–host metabolic interactions

Beyond directly metabolizing oxalate, gut microbes produce a myriad of metabolites that can influence stone formation indirectly by modifying the host’s metabolic and immune environment. A few key microbe-derived factors have emerged in recent studies:

Short-Chain Fatty Acids (SCFAs): Produced by fermentation of dietary fibers by gut bacteria (mainly in the colon), SCFAs such as acetate, propionate, and butyrate have systemic effects. SCFAs lower colonic pH and can strengthen the gut epithelial barrier. A lower colonic pH can reduce passive oxalate absorption (since more oxalate remains protonated as insoluble oxalic acid) (Wang et al., 2021). Moreover, SCFAs like butyrate serve as fuel for colonocytes, promoting tight junction integrity and reducing intestinal permeability. If gut permeability is compromised (as can happen in dysbiosis), it could lead to increased translocation of microbial products and a state of chronic inflammation. Low-grade systemic inflammation has been linked to conditions that favor stones (e.g. insulin resistance, as well as renal tubular injury that might promote crystal adherence) (Wang et al., 2021). Indeed, one hypothesis is that gut dysbiosis -> reduced SCFAs -> leaky gut -> endotoxemia -> inflammatory milieu in kidneys -> enhanced crystal retention. Supporting this, patients with recurrent stones have shown evidence of inflammatory activation and oxidative stress, which might be exacerbated by endotoxins from the gut. Thus, maintaining SCFA-producing flora may confer protection not only by local gut effects but also by dialing down systemic inflammation and favorably influencing urinary inhibitors (some studies suggest SCFAs can increase urinary citrate excretion) (Wang et al., 2025; Zalewska-Piątek et al., 2025). In addition to these general barrier- and metabolism-related effects, SCFAs may also act through SCFA-sensing G-protein-coupled receptors, particularly GPR43, to influence the gut-kidney axis in calcium oxalate nephrolithiasis. Among these pathways, GPR43 has received the strongest mechanistic support in experimental stone models. Jin et al. showed that SCFA supplementation attenuated glyoxylate-induced calcium oxalate crystal deposition through a GPR43-dependent immunomodulatory mechanism, indicating that SCFA signaling can suppress pro-inflammatory responses that favor crystal retention and renal injury (Jin et al., 2021). Related experimental work has also suggested that SCFAs may reduce stone burden by regulating intestinal oxalate transport, including modulation of SLC26A6 expression, thereby linking microbial fermentation products not only to immune homeostasis but also more directly to oxalate handling (Liu et al., 2021). Taken together, these findings support the view that the protective effects of SCFAs are not limited to nonspecific improvements in gut health, but may involve a mechanistically relevant SCFAs-GPR41/43 signaling axis that connects microbial metabolism, host inflammatory responses, intestinal oxalate transport, and downstream calcium oxalate lithogenesis (Jin et al., 2021; Liu et al., 2021).

Bile Acids: Primary bile acids are synthesized in the liver and released into the gut to aid digestion. Gut microbes convert primary bile acids into secondary bile acids (via deconjugation and other modifications). Disruption in the gut microbiota can skew bile acid profiles. A recent mouse study by Liu et al. (2025) showed that gut microbiota dysbiosis led to an accumulation of certain bile acid species which, upon entering the circulation, caused kidney tubular injury and promoted calcium oxalate crystal deposition in the kidneys (Liu et al., 2025a). Specifically, an excess of secondary bile acids was implicated in damaging renal epithelial cells, creating a nidus for crystals – a condition the authors termed a “microbiota–bile acid–oxalate axis” in stone pathogenesis (Liu et al., 2025a). Modulation of the gut microbiota in mice using antibiotics or probiotics was shown to alter bile acid pools and correspondingly influence stone burden (Liu et al., 2025a). This reveals a novel mechanism wherein an imbalance perturbs bile acid metabolism, indirectly affecting the kidney’s susceptibility to stone formation. It also raises the possibility that therapies targeting bile acid profiles (e.g. bile acid sequestrants or probiotics that modulate bile-metabolizing bacteria) might mitigate stone risk in certain contexts (Chen et al., 2021).

Unconjugated Bilirubin: Another intriguing metabolite comes from bilirubin metabolism. Li et al. (2025b) discovered that gut microbiota dysbiosis can increase levels of unconjugated bilirubin in the host, which then can deposit in the kidney and serve as a nidus or promoter for calcium oxalate crystallization (Li et al., 2025b). Bilirubin is a breakdown product of hemoglobin normally conjugated in the liver and excreted in bile. Certain gut bacteria can deconjugate bilirubin or alter its recycling. In a dysbiotic state, excess unconjugated bilirubin may accumulate systemically. Li et al. (2025b) showed that by correcting the gut microbiota (using interventions that reduced dysbiosis), systemic bilirubin levels dropped, and renal crystal deposition decreased in their animal model (Li et al., 2025b). This suggests that microbiome-regulated pigment metabolism (i.e. bilirubin) is another axis linking gut health to kidney stone formation. Unconjugated bilirubin in the kidney may act as a crystal nucleation surface or may impair local antioxidant defenses, facilitating crystal retention. While this mechanism is newly identified and needs validation in humans, it expands our view of how extensively the microbiome’s metabolic reach can extend – from classic metabolites like SCFAs to unexpected ones like pigments.

Immune Modulation and Epithelial Integrity: Gut microbes constantly interact with the host immune system. Dysbiosis often leads to an increase in pro-inflammatory microbial products (e.g. LPS, peptidoglycan) reaching the circulation, which can induce a state of chronic inflammation. Stone formation has been associated with immune responses in the kidney – for example, macrophage infiltration and the release of inflammatory cytokines that promote Randall’s plaque formation or crystal adhesion (Wang et al., 2021). A dysbiotic gut could contribute to a primed immune system that exaggerates these responses. Conversely, when a stone forms and causes renal injury, damage-associated molecular patterns (DAMPs) and cytokines from the kidney can travel to the gut and increase intestinal permeability or alter the microbiota – a two-way feedback loop (Wang et al., 2021). Recent perspectives describe this as a “gut–kidney inflammatory axis”: gut-derived inflammation makes the kidney more hospitable to stones, and kidney injury feeds back to disrupt gut barrier function, further worsening hyperoxaluria. One practical example is that after bariatric surgery (which is known to increase stone risk via hyperoxaluria), patients show both an altered microbiome and markers of intestinal inflammation, suggesting the gut–kidney axis disturbance is at play. In short, microbial metabolites that influence immune signaling (like LPS) and those that maintain epithelial health (like butyrate) are critical. Their imbalance in dysbiosis ties into the pathophysiology of stones beyond just oxalate concentration (Xu et al., 2024b; Chmiel et al., 2022).

In summary, microbe–host metabolic interactions create a complex network influencing stone formation. Although oxalate degradation represents the most direct microbial link to calcium oxalate nephrolithiasis, a broader spectrum of microbial metabolites, including SCFAs, bile acids, unconjugated bilirubin, and other bioactive compounds, together with immune modulation and epithelial barrier integrity, also contributes to the lithogenic environment. These findings raise the possibility that targeting discrete metabolic pathways, such as bile acid dysregulation or unconjugated bilirubin accumulation, may offer a more precise therapeutic strategy than broad-spectrum probiotic supplementation in selected contexts. In principle, pathway-directed interventions could be advantageous when a specific lithogenic metabolite is mechanistically implicated, because they may directly interrupt a defined host–microbe metabolic axis rather than broadly reshaping the microbial community. However, the current evidence supporting bile acid- or bilirubin-targeted approaches is still largely derived from mechanistic and animal studies, whereas clinical data remain unavailable. Broad-spectrum probiotics, although less specific, have the practical advantages of relative safety, accessibility, and the potential to influence multiple pathways simultaneously, but their effects in human stone disease remain heterogeneous and often modest. At present, the available literature does not allow a definitive conclusion that targeting these specific metabolic pathways is more effective than probiotic supplementation in clinical practice. Instead, these strategies should be viewed as potentially complementary, with pathway-targeted approaches representing a promising direction for precision prevention that requires validation in well-designed human studies. Overall, the application of metabolomics and metatranscriptomics in stone research continues to illuminate these interconnected pathways and reinforces the view that kidney stone disease is not merely a physicochemical disorder, but also a systemic metabolic and inflammatory condition shaped in part by the gut microbiota.

### 6.3 Findings from multi-omics studies

To fully capture the gut–kidney microecological changes in nephrolithiasis, researchers have turned to multi-omics approaches, integrating data from metagenomics, metatranscriptomics, and metabolomics (and even proteomics). These holistic studies provide a systems-level view of how the microbiome in stone patients differs not just in composition, but in functional potential and actual activity.

One landmark study by Al et al. (2023) performed extensive 16S rRNA gene sequencing and shotgun metagenomics on a large cohort of kidney stone formers vs controls (Al et al., 2023). They confirmed that stone formers have significantly reduced gut microbiota α-diversity and notable taxonomic shifts, but more importantly showed that the functional gene repertoire of the microbiome was altered. Pathways for oxalate degradation were, as expected, less abundant in stone patients’ metagenomes (consistent with loss of *O. formigenes* and lactobacilli) (Al et al., 2023). In parallel, genes associated with virulence and stress responses were relatively enriched. Network analysis revealed that the healthy control microbiomes had a robust, interconnected community structure, whereas stone patients’ microbiomes showed a fragmented network with less cooperative interaction among microbes (Al et al., 2023). This indicates a breakdown of the normal ecological network stability in dysbiosis, which might make the system more vulnerable to perturbations (like diet changes or antibiotics) and less resilient in maintaining oxalate homeostasis.

Other studies have combined metatranscriptomics (microbial gene expression) with metabolomics to link microbial function to metabolite outcomes. For example, in one analysis, researchers sequenced fecal microbial mRNA and measured fecal metabolites in hyperoxaluric stone patients (Bacchetta and Lieske, 2022; Liu et al., 2020). They found that transcripts of oxalate-degrading enzymes were indeed lower in patients, yet what stood out were widespread alterations in other metabolic pathways: stone formers showed downregulation of genes for beneficial metabolites (like butyrate biosynthesis) and upregulation of genes related to oxidative stress and biofilm formation (Bacchetta and Lieske, 2022; Liu et al., 2020). Correspondingly, their metabolomic profile showed deficiencies in protective metabolites (SCFAs, certain amino acid derivatives) and accumulations of potentially harmful ones (markers of oxidative stress, gut-derived urates, etc.) (Liu et al., 2020). This suggests that the microbiome’s contribution to stone risk extends beyond oxalate itself, involving a broader dysmetabolism. In other words, a stone-prone microbiome is not simply one that fails to eat oxalate; it is one that broadly fails to perform its normal symbiotic functions (like producing vitamins and anti-inflammatory compounds) and may even produce excess of metabolites that promote stone pathology (like pro-oxidants or metabolites that injure the gut or kidney lining).

Multi-omics studies have also attempted to integrate multiple body sites. For instance, one study profiled the microbiota in the gut, urine, and on the surface of extracted stones from the same patients (Al et al., 2023). The authors reported that concordant dysbiosis was present in all sites: the stone patients had distinct gut and urine microbiomes compared to controls, and interestingly, some of the bacterial DNA found in stone matrices corresponded to genera detected in the patients’ urine and stool (Al et al., 2023). This raises the hypothesis that bacteria from the gut could translocate (perhaps via the bloodstream) to the kidney and become embedded in stones. While speculative, it is known that small numbers of gut bacteria can enter circulation (especially in conditions of increased gut permeability) and potentially seed distant sites. Another multi-site approach by Denburg et al., 2020 looked at pediatric cases and found that children with stones had both gut dysbiosis and altered urinary metabolomic profiles, such as low urinary citrate; intriguingly, lower gut microbial diversity correlated with lower urine citrate excretion (Bacchetta and Lieske, 2022). Citrate is a key inhibitor of stone formation, and its excretion can be influenced by systemic acid-base status which, in turn, is partly modulated by SCFAs and other gut-derived factors. The multi-site evidence therefore strengthens the concept of a systemic microecological imbalance in stone disease, rather than an isolated gut or isolated kidney effect (Denburg et al., 2020).

Finally, researchers are identifying specific molecular players at the microbe–stone interface. For example, bacteria in the urinary tract can produce extracellular polymeric substances that form a biofilm scaffold on Randall’s plaques (interstitial apatite plaques in renal papillae that often serve as anchors for calcium oxalate stones) (Jung et al., 2023). Scanning electron microscopy has visualized bacterial imprints on Randall’s plaque surfaces, and culture of stone fragments sometimes yields bacteria even when urine cultures are negative, indicating bacteria sheltered in biofilms (Jung et al., 2023). E. coli and other Gram-negatives can release outer membrane vesicles (OMVs) loaded with adhesins and toxins; these OMVs have been shown to bind to calcium crystals and promote their aggregation into larger stones (Wang et al., 2021). On the flip side, some bacteria produce inhibitors: *Lactobacillus* species can secrete lactic acid and acetic acid, which lower urine pH locally and can chelate calcium, and even induce the host to excrete more citrate, a natural crystallization inhibitor (Agudelo et al., 2024a). Another example is the production of an enzyme like oxalate decarboxylase (OxDC) by certain gut microbes; if present in the kidney (by colonization or via circulation), OxDC could potentially degrade oxalate *in situ*. One study noted an imbalance of OxDC-producing bacteria in antibiotic-treated stone model rats and proposed supplementing factors (like zinc) to boost microbial OxDC activity as a way to reduce stones (Li et al., 2025b). These mechanistic details are still being unraveled, but they illustrate the emerging view of a “microbe–crystal–host” interaction network at the microscopic level: bacteria and their products can adhere to crystals or tubule surfaces, altering crystallization kinetics, and the host’s immune response can either clear these microbe-crystal complexes or, if dysregulated, inadvertently aid stone growth by causing granulomatous deposits (Xiang et al., 2022).

In sum, multi-omics and mechanistic studies portray a highly complex but increasingly coherent picture: kidney stone formation is influenced by a web of microbial and metabolic interactions. Key features include loss of oxalate-degrading capacity, loss of beneficial metabolite production, increased pro-inflammatory and crystal-adhering factors, and breakdown of healthy network interactions in the microbiome. These insights not only improve our understanding of pathophysiology but also point to potential targets for intervention – for example, boosting SCFA producers, blocking harmful microbial metabolites, or preventing bacterial crystal adhesion. The hope is that with continued multi-omics research, we can identify specific microbiome signatures or metabolites as biomarkers for stone risk, and novel therapeutic targets to break the chain of events linking dysbiosis to stone pathogenesis.

## 7 Microbiome-based interventions and clinical prospects

### 7.1 Dietary modulation

Diet is a cornerstone of kidney stone management, and it also represents a major lever for altering the gut microbiota. Dietary components can influence stone risk both directly (by changing urinary solute excretion) and indirectly (by shaping the gut microbial community). Modern Western diets – high in animal protein, fat, sugar, and low in fiber – are associated with increased nephrolithiasis incidence (Balawender et al., 2024). Such diets tend to acidify the urine (from high protein), raise calcium and oxalate excretion (from high salt and high oxalate intake respectively), and reduce urinary citrate (citrate excretion drops with acid load and low fruit intake) (Balawender et al., 2024). Simultaneously, a Western diet can promote gut dysbiosis, reducing beneficial bacteria that thrive on fiber and increasing those that prefer simple sugars and animal fats. This combination is thought to be deleterious for kidney stone risk (Chamberlain et al., 2020).

Key dietary factors include Oxalate intake – diets rich in oxalate (found in spinach, nuts, chocolate, certain vegetables) can significantly raise urinary oxalate, especially if calcium intake is low. A paradox exists where low calcium diets actually increase stone risk, because dietary calcium binds oxalate in the gut; insufficient calcium leaves freer oxalate to be absorbed (Balawender et al., 2024). Thus, dietary advice for CaOx stone formers is to avoid oxalate gluttony but still consume moderate calcium (preferably with meals to bind oxalate). Animal protein – high intake increases acid load and urinary calcium and reduces citrate; it may also increase gut colonization by certain bile-tolerant bacteria at the expense of fiber fermenters. Fiber and prebiotics – a high-fiber diet is generally beneficial; fiber fermentation yields SCFAs which, as discussed, may protect against stones via several mechanisms. Additionally, fiber can increase stool bulk and frequency, potentially entrapping more oxalate for fecal excretion.

Recent research emphasizes personalized nutrition based on microbiome, metabolic, and potentially host genetic profiles. An additional layer of complexity is that the response to dietary intervention may also depend on host genetic variation in oxalate transport and handling. In particular, SLC26A6 plays a central role in intestinal oxalate secretion, and experimental studies have shown that impaired transporter function can promote hyperoxaluria and calcium oxalate stone formation. This raises the biologically plausible possibility that inter-individual variation in oxalate transport pathways could modify the effectiveness of dietary strategies such as low-oxalate intake or high-fibre, microbiome-supportive diets. However, the studies reviewed here did not provide sufficient direct evidence to determine whether specific host genetic variants, including SLC26A6-related variation, consistently predict differential dietary response in clinical stone formers. Nevertheless, current work has already begun to support a more individualized dietary approach in stone prevention. Balawender et al. (2024) argue for individualized dietary therapy in stone patients by integrating dietary habits, urinary chemistry, and gut microbiota features to tailor interventions (Balawender et al., 2024; Wang et al., 2023).

One innovative concept is the Dietary Index for Gut Microbiota (DI-GM), developed in a 2025 study using National Health and Nutrition Examination Survey (NHANES) data (Wang et al., 2025). The DI-GM is a score reflecting how conducive a person’s diet is to foster a healthy gut microbiota (rich in plant fibers, variety, fermented foods, etc.). Wang et al. found that a higher DI-GM score correlated with a significantly lower prevalence of kidney stones: each standard deviation increase in DI-GM was associated with ~22% reduced odds of having a history of stones (odds ratio (OR) ~0.78) (Wang et al., 2025). The protective association was especially strong in women, African Americans, and individuals with a history of high alcohol intake (Wang et al., 2025). The hypothesized reason is that microbiome-friendly diets (high DI-GM) increase SCFA production and gut microbial diversity, which can lead to higher urinary citrate and lower inflammation, as well as possibly lower oxalate absorption (Wang et al., 2025). Diet patterns like the DASH diet (Dietary Approaches to Stop Hypertension), which is high in fruits, vegetables, and fiber, have in fact been linked to lower kidney stone risk in epidemiologic studies, aligning with the microbiome mechanism (Pei et al., 2025; Junier et al., 2025; Pebenito et al., 2019b).

It is important to note that current dietary interventions for stones (hydration, low salt, moderate animal protein, ample fruits/veggies) already incidentally promote a healthier microbiome. Thus, diet represents a practical means of modulating the gut–kidney axis in stone prevention. Current evidence most consistently supports plant-rich, high-fiber dietary patterns as microbiome-compatible strategies that may help reduce lithogenic risk (Balawender et al., 2024; Wang et al., 2025; Stone, 2022).

### 7.2 Probiotics and synbiotics

Given the evidence implicating specific bacteria in oxalate metabolism, a logical therapeutic approach is probiotic supplementation – introducing beneficial microbes to correct dysbiosis or boost oxalate degradation. Early interest in this area dates to attempts to colonize patients with *Oxalobacter formigenes* as a probiotic. While *O. formigenes* capsules showed some success in colonizing the gut, clinical outcomes (urinary oxalate reduction, stone recurrence prevention) were inconsistent (Bostanghadiri et al., 2021). The challenges of *O. formigenes* (obligate anaerobe, slow growth, antibiotic susceptibility) led researchers to explore other probiotic strains that are easier to handle yet can degrade oxalate.

One avenue has been the screening of lactic acid bacteria from foods and other ecological sources for oxalate-degrading ability. Recent literature has highlighted the growing translational interest in oxalate-degrading lactic acid bacteria, not only as conventional probiotic candidates but also as potential functional starters for fermented foods and related microbiome-supportive applications (Al-Kabe and Niamah, 2024). Continued screening efforts have further suggested that selected lactic acid bacterial isolates may combine oxalate-degrading activity with broader probiotic properties, supporting their possible future development as targeted adjuncts for oxalate management (Al-Kabe and Niamah, 2025). In this context, it is also important to acknowledge the post-2020 taxonomic reclassification of the former genus *Lactobacillus* into 23 genera, because several reclassified genera, particularly *Lactiplantibacillus, Lacticaseibacillus*, *Limosilactobacillus*, and related taxa, have been investigated for oxalate-degrading capacity and probiotic potential (Al-Kabe and Niamah, 2024, 2025). Xu et al. (2024a) isolated dozens of lactic acid bacterial strains from fermented foods and screened them for oxalate degradation *in vitro*. They identified a strain of *Lactiplantibacillus plantarum* (AR1089) with high oxalate-degrading capacity, with approximately 14–17% oxalate degraded *in vitro* (Xu et al., 2024a). When administered to rats with hyperoxaluria and CaOx stone induction, *Lactiplantibacillus plantarum* AR1089 significantly reduced renal calcium oxalate crystal deposition, lowered urinary oxalate levels, and ameliorated oxidative stress markers compared with control stone-forming rats (Xu et al., 2024a). Kidney injury markers (KIMs) (KIM-1, osteopontin, etc.) were also downregulated by the probiotic, and gut microbiota analysis suggested AR1089 supplementation increased overall diversity and beneficial genera (Xu et al., 2024a). Similarly, another group tested a multi-strain commercial probiotic mix in a rat model of hyperoxaluria and reported reduction in renal crystal deposition and oxidative damage in the kidney (Liu et al., 2025b). Although that model was primarily one of hyperuricemia (using adenine to induce both hyperuricemia and some oxalate elevation), the probiotic’s benefits were attributed in part to enhanced oxalate metabolism and reduction of gut and renal oxidative stress (Liu et al., 2025b; Devarajan, 2018; Yang et al., 2025b, 2025; Zhou et al., 2025; Chamberlain et al., 2019; Miller et al., 2017a; Liu et al., 2025c).

However, human trials of probiotics for stone prevention have yielded mixed outcomes. Some small trials using lactic acid bacterial or Bifidobacterium-based supplements did not show a significant reduction in urinary oxalate, possibly because these strains had limited oxalate-degrading ability or could not colonize well (Bostanghadiri et al., 2021). A 2021 review noted that while *in vitro* and animal data are promising, clinical evidence is not yet robust and any benefits may depend on strain selection and dosing (Bostanghadiri et al., 2021). Examples include O. formigenes itself or engineered bacteria, as discussed below in the section on emerging live biotherapeutic strategies (Cao et al., 2022; Suryavanshi et al., 2016; Sidhu et al., 1998).

The concept of synbiotics (combined probiotic + prebiotic) is also being explored. A synbiotic can provide a tailored substrate to support the colonization and activity of the administered probiotic. In 2025, Li X-M et al. reported that a novel synbiotic formulation effectively prevented calcium oxalate stone formation in rats by “restoring gut microbiota homeostasis” (Li et al., 2025c). The success of synbiotics would not be surprising, as the gut environment often needs both the right microbes and the right food for those microbes.

Despite the promise, challenges remain for probiotic therapy: ensuring the bacteria survive gastric transit, successfully engraft in the competitive gut ecosystem, and actually perform the desired function *in vivo*. Colonization resistance can be strong; many orally taken probiotics transiently pass through without permanent residency. Safety is another consideration – probiotics are generally safe, but in immunocompromised patients there’s a small risk of bacteremia. For stone patients, however, probiotics have been used without major issues in trials to date.

Overall, probiotics and synbiotics remain promising but not yet established strategies in nephrolithiasis, with current evidence supporting preclinical efficacy more consistently than clinical benefit (Miller et al., 2016).

### 7.3 Fecal microbiota transplantation

FMT – the transfer of stool from a healthy donor to a patient to re-establish a balanced microbiome – has gained fame for treating *Clostridioides difficile* infection and is being investigated in various metabolic and inflammatory conditions. The rationale in kidney stones is that since stone formers have global gut dysbiosis, resetting the gut microbiota to a healthy state might reduce stone propensity. Rather than adding one or two strains (as with probiotics), FMT aims to overhaul the entire microbial community.

Preclinical studies provide initial support for FMT in nephrolithiasis. An *in vivo* study by An et al. (2025) demonstrated that transplanting fecal microbiota from healthy rats into rats with calcium oxalate stones led to a significant reduction in stone formation (An et al., 2025). Analysis showed that FMT recipients had their gut microbiota diversity restored and, importantly, a resurgence of oxalate-degrading bacteria like *Oxalobacter* and *Lactobacillus* that had been depleted in the stone model (An et al., 2025). Additionally, levels of inflammatory mediators in the gut and kidney were reduced after FMT, indicating a systemic anti-inflammatory effect (An et al., 2025).

At present, however, the evidence base for FMT in nephrolithiasis remains very limited. Although preclinical animal studies suggest potential benefit, published clinical studies specifically evaluating FMT for kidney stone prevention or treatment are currently lacking (Bostanghadiri et al., 2021). Accordingly, its clinical relevance in nephrolithiasis remains speculative and has not yet been supported by robust human evidence.

Of course, FMT comes with practical and regulatory challenges. It involves screening donors for pathogens, preparing stool material (usually via enema, nasal duodenal tube, or capsules), and it carries a small risk of transmitting infections or causing GI upset. There is also the uncertainty of how long the transplanted microbiota will persist; frequent antibiotic exposures could erase gains from FMT.

In summary, although FMT remains of mechanistic interest, it should currently be regarded as an experimental and early-stage concept in nephrolithiasis rather than a near-term clinical option. Before any broader application can be considered, well-designed clinical studies will be required to determine whether FMT can safely and effectively reduce urinary oxalate or prevent stone recurrence. Given the current lack of human evidence, its clinical prospects in nephrolithiasis remain limited (Ticinesi et al., 2018).

### 7.4 Emerging engineered bacterial therapies

A major emerging frontier is the use of synthetic biology to create engineered bacteria that perform therapeutic functions in the gut. In the context of oxalate metabolism, researchers have succeeded in programming microbes with enhanced oxalate-degrading capabilities and control features. A groundbreaking example is the study by Whitaker et al. published in *Science* in 2025 (Whitaker et al., 2025). The team engineered a human commensal bacterium (*Phocaeicola vulgatus*, formerly classified in the Bacteroides genus) by introducing an entire five-gene pathway for oxalate degradation into it (Whitaker et al., 2025). This gave the strain a robust ability to break down oxalate. Additionally, they incorporated genetic circuits to control the bacterium’s colonization: the engineered strain was designed to only thrive in the presence of a specific polysaccharide (porphyran) that is not common in the human diet unless supplemented. Porphyran acts as a “selective fuel” for the microbe. This means the bacteria can be selectively engrafted and also de-engrafted based on porphyran administration – a safety feature for reversible colonization (Whitaker et al., 2025).

In preclinical tests, oral administration of this engineered *P. vulgatus* to hyperoxaluric mice significantly reduced urinary oxalate levels and prevented CaOx crystal accumulation (Whitaker et al., 2025). Essentially, the bacteria in the gut took up oxalate and degraded it before it could reach the kidneys. A notable aspect of the study was the accompanying Phase 1/2a clinical evaluation in human volunteers. In the trial, subjects with mild hyperoxaluria were given the engineered bacteria orally, along with varying doses of porphyran to facilitate engraftment (Whitaker et al., 2025). The results were promising: the engineered strain successfully colonized the colons of participants in a dose-dependent manner (more porphyran = greater colonization), and colonization was reversible (when porphyran was stopped, the bacterial population declined) (Whitaker et al., 2025). Importantly, those who achieved colonization showed a reduction in urinary oxalate excretion compared to baseline (Whitaker et al., 2025). This provides the first proof in humans that a designer probiotic can safely modulate oxalate metabolism and potentially treat hyperoxaluria.

There were challenges noted – for instance, some issues with the long-term stability of the oxalate-degrading genetic pathway in the bacteria (a few mutations arose over time), indicating that further optimization is needed for durability (Whitaker et al., 2025). However, the concept has been validated: live biotherapeutic agents (LBPs) can be created to target kidney stone risk factors (Whitaker et al., 2025; Hunthai et al., 2024).

Although these findings provide an important proof-of-concept, regulatory pathways for live biotherapeutic products are still evolving, and larger studies will be needed to determine whether such approaches can meaningfully reduce clinical stone outcomes beyond urinary biochemical markers (Xie et al., 2024). Overall, engineered bacterial therapies illustrate how mechanistic insights into the gut–kidney microbiome–oxalate axis are beginning to translate into functionally targeted interventions, although their clinical role in nephrolithiasis remains early-stage.

### 7.5 Antibiotic stewardship and other strategies

While adding “good” microbes or diets is one side of the coin, another important aspect is avoiding practices that harm the microbiome. Antibiotic stewardship has emerged as a pertinent strategy for kidney stone prevention. As discussed, epidemiological studies have linked systemic antibiotic exposure to increased risk of nephrolithiasis, likely through depletion of oxalate-degrading bacteria and other dysbiosis effects (Nazzal et al., 2021; Tasian et al., 2018). For instance, exposure to broad-spectrum penicillins was associated with 27% higher odds of stones, and even higher odds for sulfa drugs (~2.3-fold) in the months following antibiotic use (Tasian et al., 2018). Considering this, experts have suggested that judicious use of antibiotics – particularly in children and individuals at risk for stones – could reduce stone incidence (Liu et al., 2025b; Nazzal et al., 2021). If antibiotics must be used, subsequent microbiome-supportive measures such as diet optimization or selected probiotics may be considered to help restore lost oxalate-degrading capacity.

Another auxiliary strategy concerns adjunct therapies that have indirect microbiome or antioxidant effects. For example, certain herbal extracts are being studied in stone models for their nephroprotective and microbiome-modulating properties. Zhou et al. (2022) tested daphnetin, a plant-derived compound, in a rat hyperoxaluria model and found it had a nephroprotective effect – reducing oxidative injury and crystal deposition, possibly by altering gut microbiota composition and boosting endogenous antioxidant defenses (Zhou et al., 2022). Some traditional supplements for stone prevention may also interact with the gut microbiota. For example, a recent clinical study in uric acid stone patients reported that potassium sodium hydrogen citrate intervention was accompanied by changes in gut microbiota composition and short-chain fatty acid profiles, alongside improvement in urinary pH and stone-related clinical features (Cao et al., 2024).

A notable mention is the role of alkali supplementation (citrate/bicarbonate) in stone formers with low urine citrate or acidic urine. These supplements improve urinary chemistry by raising citrate (inhibiting CaOx and CaP stone formation) and alkalinizing urine (preventing uric acid stones). Hydration remains critical too – high fluid intake dilutes urine and is the most proven way to cut recurrence risk.

**Figure 4:**
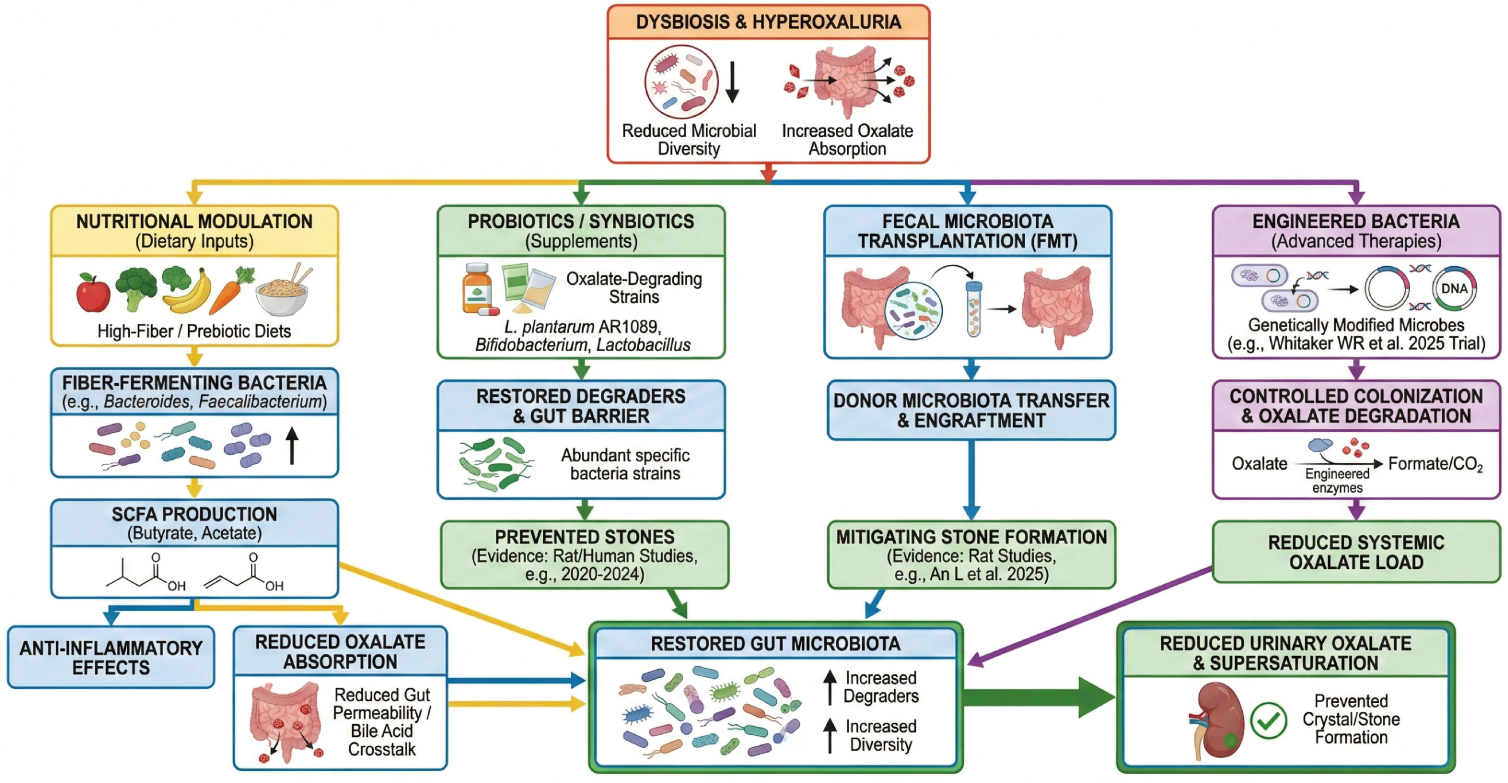
Flowchart of microbiome-based interventions for calcium oxalate nephrolithiasis. The flowchart illustrates microbiome-based interventions for calcium oxalate nephrolithiasis, starting from dysbiosis/hyperoxaluria through nutritional modulation (high-fiber/prebiotic diets promoting SCFA production), probiotics/synbiotics (oxalate-degrading strains like L. plantarum AR1089), FMT (e.g., An et al., 2025), and engineered bacteria (e.g., Whitaker et al., 2025), converging on restored microbiota, reduced urinary oxalate, and prevented stone formation via bile acid crosstalk and inflammation reduction.

In summary, preservation of the endogenous microbiota is an important complement to microbiome-restorative strategies, and avoidance of antibiotic-induced dysbiosis may represent a practical preventive consideration in stone disease. **Figure 4** provides an overview of this stepwise intervention pathway, starting from dysbiosis and hyperoxaluria and converging on restored gut microbial diversity, reduced oxalate burden and prevention of crystal/stone formation.

### 7.6 Clinical translation and guideline implications

At present, insights into the gut–kidney microbiome–oxalate axis refine rather than replace guideline-based care. Current stone guidelines still prioritize high fluid intake, dietary sodium and animal-protein restriction, adequate calcium and targeted pharmacotherapy, but these measures can be framed in a microbiome-informed way, for example by emphasising plant-rich, high-fibre dietary patterns and prudent antibiotic use in recurrent CaOx stone formers. Probiotics or synbiotics may be discussed as low-risk, experimental adjuncts in selected patients, whereas faecal microbiota transplantation and engineered live biotherapeutics should remain confined to clinical trials. In the longer term, it is plausible that future stone guidelines will incorporate microbiome-supportive dietary advice, recognise antibiotic exposure and gut dysbiosis as modifiable risk factors, and eventually consider validated microbiome or metabolomic signatures for risk stratification and selection of candidates for microbiome-targeted therapies (Li et al., 2025a).

## 8 Conclusion and future perspectives

### 8.1 Overall quality of evidence and methodological limitations

Overall, the body of evidence synthesized in this review remains low-to-moderate in quality when judged against conventional evidence hierarchies. Most human data derive from case–control or cross-sectional studies and small single-center cohorts with limited follow-up, whereas adequately powered randomized trials and large prospective cohorts remain rare. Consequently, many reported associations between microbial taxa, pathways, metabolites, and stone phenotypes should still be interpreted as hypothesis-generating rather than causal.

A second limitation is the pervasive reliance on surrogate endpoints. Many microbiome-targeted interventions in stones focus on intermediate outcomes such as changes in urinary oxalate excretion, microbiome composition or circulating metabolites, while few trials have stone recurrence, stone burden or time-to-event outcomes as primary endpoints. Follow-up durations are often measured in weeks to a few months rather than years, making it difficult to ascertain whether short-term biochemical or microbiome shifts translate into durable reductions in clinical events.

Substantial methodological heterogeneity also exists in microbiome assessment. Studies differ in sampling sites (stool, midstream urine, catheterized urine, or stone surface), DNA extraction protocols, sequencing platforms (16S rRNA gene versus shotgun metagenomics), read depth, reference databases, bioinformatic pipelines and statistical workflows. In addition, the extremely low biomass of the urinary microbiota makes these datasets particularly susceptible to environmental contamination and batch effects, which can either amplify spurious signals or obscure true biological associations if not rigorously controlled.

The present review has its own limitations. Although we implemented a structured search across PubMed/MEDLINE, Embase, and Web of Science for the period 2010–2025, this work remains a narrative rather than a fully systematic review; publication bias and language restrictions may have led to underrepresentation of negative or non-English studies. No formal risk-of-bias assessment was undertaken, and we did not grade the certainty of evidence using frameworks such as Grading of Recommendations Assessment, Development and Evaluation (GRADE). However, to improve methodological transparency, we clarified the eligibility framework and added a PRISMA-style flow diagram summarizing the study selection process. Furthermore, our synthesis centers on 104 references selected for their relevance to oxalate metabolism, the gut–kidney axis, and key mechanistic or translational developments in the field. While this strategy allows in-depth discussion of key mechanistic themes, it may underweight some peripheral but relevant lines of research, and the rapid pace of microbiome and synthetic-biology studies means that new evidence is likely to emerge that could refine or challenge parts of the conceptual framework presented here.

Over the past decade, the field has shifted from viewing nephrolithiasis primarily through a physicochemical lens to recognizing the contribution of host–microbe interactions to lithogenesis. We now recognize that kidney stone formers often exhibit a convergence of factors: gut microbiota alterations (loss of oxalate degraders, reduced diversity, metabolite imbalances), urinary microbiota changes (presence or absence of bacteria influencing crystallization), and systemic metabolic/inflammatory perturbations, all of which interweave in stone pathophysiology. Multi-omics studies have mapped out this complex network, providing molecular evidence that goes beyond simple correlations.

Against this background, several key questions remain for future research. First, much of the evidence linking the microbiome to stones is *associative*. We know stone patients have dysbiosis, but causality is not fully proven. Does dysbiosis drive stone formation, or do recurring stones and associated treatments drive dysbiosis (or is it a bit of both, forming a vicious cycle)? Longitudinal cohort studies and intervention trials will be crucial to establish directionality.

Another area needing exploration is the urinary microbiota’s consistency and significance. Findings about the role of the urinary microbiota in stones are still preliminary. Some studies conflict, and contamination issues cloud interpretation. Standardization in sampling and analysis is needed so that results from different centers can be compared. The influence of the urinary microbiota may prove to be more moderate than that of the gut microbiota, but even a moderate effect could still be clinically relevant.

From a translational perspective, dietary modulation is currently the most immediately applicable microbiome-relevant strategy, whereas probiotics remain investigational, FMT is experimental, and engineered live biotherapeutics are still at an early developmental stage.

Large-scale metagenomic studies across diverse populations could identify a “stone microbiome signature” that might serve as a biomarker for risk stratification. In our view, the most critical missing data point for clinical translation is a prospectively validated biomarker signature that integrates microbiome composition, microbial metabolites, and host determinants to predict clinically relevant stone outcomes at the individual level. Such a signature would need to be reproducible across cohorts and sufficiently robust to support risk stratification, early identification of vulnerable individuals, and selection of candidates for microbiome-targeted interventions. Additionally, exploring the interaction between host genetics and the microbiome through genome–metagenome association studies might reveal why certain people recover *O. formigenes* after antibiotic exposure whereas others do not, or why some individuals generate a more lithogenic metabolic profile. Such knowledge could support earlier identification of high-risk patients and more precise preventive strategies.

Microbial metabolites also represent plausible therapeutic targets. Future studies should determine whether pathway-targeted modulation of bile acids, SCFAs, bilirubin, or other lithogenic metabolites can deliver clinically meaningful benefit beyond broader microbiome-directed approaches.

In conclusion, nephrolithiasis should increasingly be viewed as a disorder shaped not only by host metabolism and urinary chemistry, but also by perturbed host–microbe interactions across the gut–kidney microbiome–oxalate axis. Current evidence supports a mechanistic framework linking multi-site microbiota alterations, microbial metabolites, host oxalate-handling pathways, and lithogenic injury responses, although much of this evidence remains associative and methodologically heterogeneous. Continued progress will depend on better-standardized multi-omics studies, longitudinal human cohorts, and clinical trials capable of testing whether microbiome-informed interventions can meaningfully reduce stone recurrence and other patient-relevant outcomes. By integrating current evidence into a unified host–microbe framework, this review aims to provide a conceptual basis for future mechanistic and translational work in calcium oxalate nephrolithiasis (Suarez Arbelaez et al., 2023; Kremer et al., 2025).

## Data Availability

The original contributions presented in the study are included in the article/supplementary material. Further inquiries can be directed to the corresponding authors.
